# Editorial Comment: Adverse Effects of Intravesical Onabotulinumtoxin A Injection in Patients with Idiopathic Overactive Bladder or Neurogenic Detrusor Overactivity: A Systematic Review and Meta-Analysis of Randomized Controlled Studies

**DOI:** 10.1590/S1677-5538.IBJU.2025.9902

**Published:** 2025-01-05

**Authors:** Yu Ping-Hsuan, Wang Chung-Cheng

**Affiliations:** 1 National Taiwan University En Chu Kong Hospital New Taipei Taiwan Department of Urology, En Chu Kong Hospital, College of Medicine, National Taiwan University, New Taipei City 237414, Taiwan; 2 Taipei Veterans General Hospital Department of Urology Taipei Taiwan Department of Urology, Taipei Veterans General Hospital, Taipei 112201, Taiwan;; 3 Taipei Veterans General Hospital Department of Urology Taipei Taiwan Department of Urology, College of Medicine, National Yang Ming Chiao Tung University, Taipei 112304, Taiwan;; 4 National Yang Ming Chiao Tung University Shu-Tien Urological Science Research Center Taipei Taiwan Shu-Tien Urological Science Research Center, National Yang Ming Chiao Tung University, Taipei 112304, Taiwan; 5 National Yang Ming Chiao Tung University Department of Biomedical Engineering Taoyuan Taiwan Department of Biomedical Engineering, Chung Yuan Christian University, Taoyuan 320314, Taiwan

**Toxins (Basel) 2024 Aug 5;16(8):343.**

DOI: 10.3390/toxins16080343 |ACCESS: 39195753

## COMMENT

Intravesical injection of OnabotulinumtoxinA (BTA) is an established treatment for both neurogenic detrusor overactivity (NDO) and idiopathic overactive bladder (OAB) symptoms, but it is not devoid of risks (1). The meta-analysis conducted by Yu and Wang focused on local and systemic adverse events (AE) associated with BTA injections in the bladder.

This study included 26 randomized clinical trials, 8 of which focused on NDO and 18 on idiopathic OAB. BTA versus placebo significantly increased the incidence of urinary tract infections (UTI) in individuals with NDO (relative risk, or RR, 1.54) and idiopathic OAB (RR, 2.53). The RR of urinary retention was 6.56 in the NDO and 7.32 in the idiopathic OAB group, respectively, with similar rates of de novo clean intermittent catheterization (CIC). In patients with idiopathic OAB, BTA increased the likelihood of voiding symptoms. Systemic AEs of BTA were observed in individuals with NDO, including muscle weakness (RR, 2.79) and nausea (RR, 3.15). However, the majority of systemic AEs were rare and self-limited.

These findings highlight the need of a proper alignment of patients’ expectations concerning BTA treatment, assessing the tolerability profile, as well as the stratification of risk for voiding dysfunction and urinary retention.
